# A Proposed Algorithm on the Modern Management of Rectus Sheath Hematoma: A Literature Review

**DOI:** 10.7759/cureus.20008

**Published:** 2021-11-29

**Authors:** En-Dien Liao, Yana Puckett

**Affiliations:** 1 Critical Care Medicine, Texas Tech University Health Sciences Center, Lubbock, USA; 2 Surgery, West Virginia University School of Medicine, Charleston, USA

**Keywords:** rsh, risk predictors, interventional radiology guided embolization, conservative medical management, treatment algorithm, standardization of protocol, rectus sheath hematoma

## Abstract

Rectus sheath hematoma (RSH) is a common entity with no clearly established protocol for management. Existing literature on RSH is outdated and does not incorporate modern technological advances in medicine and imaging. A total of 21 studies were included in this literature review based on PubMed and Google Scholar searches. Modern literature was selected from the last 10 years with the inclusion of three past pieces of literature. We performed a literature review to identify the latest research on RSH management and to consolidate an algorithm to help guide modern RSH treatment. Current RSH classification, scoring system, algorithm, and other predictors for treatment plan are discussed. The best RSH management requires early recognition of RSH followed by the appropriate implementation of conservative management and procedural intervention. The decision on picking the treatment of choice is assisted with the use of predictors, such as hematoma size, rate of hemoglobin drop, and the number of blood transfusions. Further studies are needed to clearly establish predictors among the different types of procedural intervention, and we hope the consolidated algorithm on current literature can help promote the standardization of protocol in the future.

## Introduction and background

Rectus sheath hematoma (RSH) is formed from a rupture of the inferior or superior epigastric artery most commonly due to traumatic or spontaneous etiology [1]. RSH is associated with nonspecific symptoms such as abdominal pain and is often unrecognized and underdiagnosed. Risk factors for RSH include anticoagulation treatment, paroxysmal coughing, hypertension, advanced age, and previous abdominal surgery [2,3].

Current first-line treatment for RSH is conservative management (CM) involving fluids resuscitation, blood replacement, reversal of anticoagulation, lab monitoring, and bed rest [2,4]. However, when CM fails, further surgical or embolization interventions are available and may be necessary. The problem lies in the lack of consensus for predictors that will clearly link a patient to a specific treatment intervention. No clear data has been shown for best predictors to support a standardized protocol due to few prospective studies on this topic. A lack of clear predictors to guide clinicians is problematic when managing patients with RSH [1]. The purpose of this study is to consolidate past and modern literature on RSH and determine a potential algorithm to manage RSH. Modern is defined as the last 10 years of research.

Methods

A total of 21 studies were included in this literature review based on PubMed and Google Scholar searches published from 1996 to 2020. Search terminologies using a combination of "rectus sheath hematoma," "management," "algorithm," "treatment," and "endovascular embolization" were completed. A discussion of various RSH management will be presented followed by a consolidated algorithm of the findings. A summary of the studies reviewed is shown in Table 1.

**Table 1:** A summary table of studies PRBC=Packed Red Blood Cell; CM=Conservative Management; IR=Interventional Radiology; Sx=Surgical Intervention; DOAC=Direct Oral Anticoagulant; CT=Computerized Tomography Scan; LMWH=Low Molecular Weight Heparin; IAP=Intra-Abdominal Pressure; RSH=Rectus Sheath Hematoma; NBCA-MS=N-Butyl Cyanoacrylate Methacryloxy Sulfolane *Balt S.A.S, Montmorency, France

Authors	Publish Year	Study Design	Sample Size	Pertinent Results
Bekirov et al. [5]	2020	Case Study	1	In a Covid-19 pneumonia case, Sx was found to be superior to IR.
Bekraki et al. [4]	2016	Case Study	1	RSH < 5 cm managed with CM, >5 cm with Sx
Berna et al. [6]	1996	Retrospective	13	CT is gold standard. Type 1-3 RSH staging
Buffone et al. [2]	2015	Retrospective	8	5 CM, 1 IR, and 1 Sx. Hemodynamic instability is a better measure of surgical need than size of hematoma.
Cakir [7]	2020	Retrospective	6	Embolization is a safe and effective treatment option for unstable RSH.
Contrella et al. [8]	2020	Retrospective	72	Scoring system based on contrast extravasation, hematoma size/volume, PRBC units transfused, and rate of Hgb drop.
Donaldson et al. [9]	2007	Case Study	3	Sx evacuation needed for unstable IAP
Gangemi et al. [10]	2017	Case Study	1	Combination of IR and Sx evacuation are successful alternatives to Sx ligation
Gradauskas et al. [11]	2018	Retrospective	29	CM had shortest hospital stay
Jawhari et al. [12]	2018	Retrospective	50	NBCA-MS embolization is safe and effective
Klingler et al. [13]	1999	Retrospective	23	Sx should be restricted for large, >5 cm hematomas or intra-abdominal bleed
Mantelas [14]	2011	Case Study	1	Ligation technique of inferior epigastric artery demonstrated immediate stabilization.
Mcbeth et al. [15]	2012	Case Study	2	IAP should be used as predictor for RSH
Onder et al. [16]	2011	Retrospective	5	RSH classification by CT. Early diagnosis and CM is key.
Ozyer [17]	2017	Retrospective	38	NBCA is highly effective and safe in hemodynamically unstable patients who failed CM.
Smithson et al. [18]	2013	Retrospective	24	LMWH are more likely to require IR than DOAC. Embolization is 1st line treatment.
Torcia et al. [19]	2017	Case Study	1	Squidperi* is safe and effective.
Tseng et al. [3]	2011	Case Study	1	Successful treatment with IR embolization despite no evidence of contrast extravasation
Villa et al. [1]	2012	Retrospective	78	CM is sufficient to manage RSH. No predictors were found.
Warren et al. [20]	2020	Retrospective	99	Shock is a predictor for PRBC. No predictors were found for IR. DOAC was not a predictor.
Yun et al. [21]	2015	Case Study	1	Sx ligation of inferior epigastric artery followed by evacuation allows good field of vision.

## Review

Conservative management

When the patient is hemodynamically stable, conservative management of RSH has been found to be the most effective treatment methodology due to the self-limiting nature of RSH [4,11,20]. Approximately 56-83% of the patients studied are successfully managed with CM alone [1,8,13,18,20]. However, that leaves between 17-44% of patients in need of intervention with no clear pathway delineated in the literature. In a 2018 retrospective study, Gradauskas et al. compared treatment modality outcomes between four cases of embolization, six cases of open surgery, eight cases of percutaneous drainage, and 10 cases of CM. They found CM had the shortest hospital stay with a mean duration of 6.7 days (SD 3.2, p=0.028) [11]. Catching RSH and implementing CM early prior to progression to shock is key [16].

Supporting the idea of CM, a larger retrospective study done by Warren et al. compared CM to other treatment outcomes. Of the 99 patients, a majority of the cases were manageable by CM alone, and only 17 and one patientsrequired interventional radiology (IR) embolization and surgical intervention, respectively [20]. Because CM was the primary treatment for the majority of RSH patients in the study, the author suggested routine surgical consults may be unnecessary [20].

Predictors for Higher Intervention

Some studies have proposed classification, scoring systems, and algorithms to serve as measurement predictors to escalate failed CM. In a historical study, hematoma size has been suggested as a prognostic factor for surgery. RSH < 5 cm in diameter can be managed with CM while large RSH > 5 cm in diameters are managed with surgical intervention [4,13]. In the historical Klingler et al. study, eight out of 23 patients had large RSH while 15 patients had small RSH. Using ultrasound, the eight large RSHs were 5-6 cm, 7-8 cm, and >9 cm with a total of one, three, and four patients corresponding to each size range, respectively [13]. Almost all of the patients with large RSH had free intraabdominal fluids while the smaller RSHs did not [13]. The study suggests the presence of free intraabdominal fluids indicates an increased likelihood to perforate [13]. Therefore, the presence of free intrabdominal fluids was linked to the increased need for surgery (p<0.0005) [13]. As a result, hematomas > 5 cm are recommended to be treated surgically [13].

Hematoma Diagnosis and Classification

Computerized tomography (CT) scan is the gold standard for RSH diagnosis followed by ultrasonography [1,2,6,11,16]. Berna et al. classified hematomas into three types. Type 1 RSH corresponds with intramuscular hematoma and minimal bleeding [6,16]. Type 2 RSH is an intramuscular hematoma with moderate bleeding, and Type 3 RSH is acute bleeding that extends outside of the muscle [6,16]. Type 1 RSH tends to resorb on its own within 30 days, and its management is mostly outpatient observation [16]. In contrast, Type 2 and Type 3 tends to resolve within 90 days, and both treatments require hospitalization along with in-hospital CM [16]. Due to hemodynamic instability, Type 3 RSH treatment requires additional blood transfusions and meticulous lab monitoring every 2-4 hours (Table 2) [16]. 

**Table 2:** Definition of the three types of RSH and their management CT=Computerized Tomography Scan; NPO=Nothing by Mouth; CBC=Complete Blood Count; RSH: Rectus Sheath Hematoma

Rectus Sheath Hematoma	Definition	Treatment
Type I	Hematoma confined within muscle with no active extravasation of contrast on CT scan and non-expanding over a period of 6 hours.	+/- Admission to hospital for observation. Observation and bedrest only.
Type II	Hematoma confined within muscle with active extravasation of contrast on CT scan.	Admission to hospital, bed rest, NPO, fluid replacement as needed, and analgesia.
Type III	Hematoma with active extravasation of contrast on CT scan that extends beyond the confines of muscle.	Admission to hospital, bed rest, NPO, fluid replacement as needed, and analgesia. May require blood transfusion for hemodynamic instability and CBC checks every 2-4 hours.

Hemodynamic Instability and Hematoma Size

Hematoma size alone may be a poor predictor for IR or surgery. In a 2015 study of eight RSH patients, five had CM, one had IR embolization, and one had surgical ligation [2]. Both the IR and surgical patients presented with hemodynamic instability upon admission while the rest had Type 2 and Type 3 hematomas [2]. Despite having Type 3 hematomas > 9 cm in diameter, the RSHs were manageable by CM alone, but the patients with hemodynamic instability were not. The author concluded hemodynamic instability to be a better predictor for higher-level intervention than the hematoma size [2]. 

Scoring System

**Figure 1:**
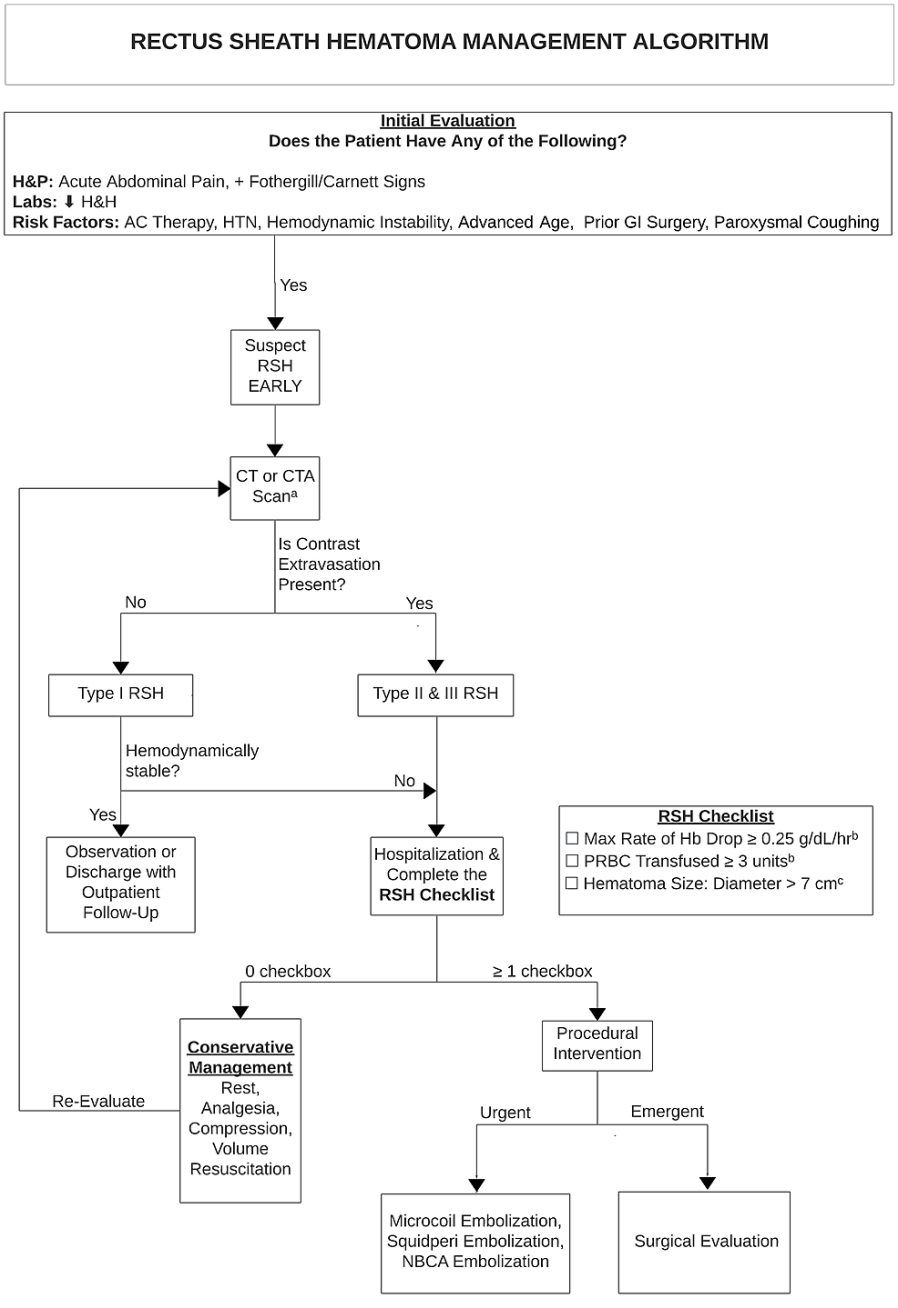
A proposed algorithm to manage rectus sheath hematoma based on a consolidation of literature review H&P=History and Physical; H&H=Serum Hemoglobin and Hematocrit; AC=Anticoagulant; HTN=Hypertension; GI=Gastrointestinal; RSH=Rectus Sheath Hematoma; CT or CTA = Computerized Tomography Scan or Computerized Tomography Angiogram; NBCA=N-Butyl Cyanoacrylate; Hb=Hemoglobin; PRBC=Packed Red Blood Cells a = When CT or CTA scan is not readily available, ultrasonography may be used as an alternative b = Contrella et al. [8] c = Klingler et al. [13]

In a recent 2020 retrospective study, Contrella et al. proposed a scoring system and algorithm. The scoring system serves as a predictor for IR embolization. Scoring is based on four criteria: the severity of active extravasation on CT scan (p=0.02), the hematoma size in liters (p=0.006), the number of packed red blood cell units transfused (p=0.03), and the rate of hemoglobin drop (p<0.0001) (Figure 1) [8]. Due to its retrospective design, the scoring system may be limited in its generalizability [8].

Anticoagulant/Antiplatelet Agent

The type of anticoagulant therapy may also serve as predictors. When compared to CM, RSH patients requiring IR embolization (80%) were more likely to receive low molecular weight heparin (p=0.047) [18]. Data on direct oral anticoagulants (DOAC) and regular heparin were insignificant [18]. In the study by Warren et al, the odds of a patient on low molecular weight heparin and needing IR embolization were high at 2.676 (p=0.08), while unfractionated heparin (1.175, p=0.774), DOAC (0, p=0.999), aspirin (0.198, p=0.039), clopidogrel (0, p=0.999), and fondaparinux (0, p=1) were low [20].

Interventional radiology embolization

When an RSH patient becomes hemodynamically unstable, further intervention is recommended. Microcoil embolization is suggested to be the first treatment option after failed CM [9,18]. One particular retrospective study reviewed five RSH patients with active contrast extravasation [7]. None of the patients had complications from the embolization, and no extravasation was found post-intervention [7]. Although one patient died, the cause of death was attributed to heart failure complications and none were related to the embolization [7]. The author concluded embolization to be a safe and effective treatment option [7].

However, potential downsides to microcoil embolization are the time-consuming, expensive, and inaccessible nature of the procedure especially during emergency cases [14]. For example, a COVID-19 case with multiple comorbidities presenting with hemorrhagic shock due to RSH was managed successfully with surgical evacuation instead of microcoil embolization [5]. Surgical evacuation was found to be superior to embolization given the time constraint during the pandemic [5]. It should be noted surgical intervention was also preferred in this situation given the patient’s comorbidities, and the intervention of choice will vary case by case.

Alternative Transcatheter Arterial Embolization

The use of n-butyl cyanoacrylate (NBCA) and methacryloxy sulfolane (MS) glue in IR is explored in place of the typical microcoil embolization intervention. NBCA offers a quicker administration compared to microcoil embolization. In a study of 50 patients who underwent NBCA-MS embolization, Jawhari et al. found a 100% technical success rate, 66% clinical success rate, 34% post-embolization recurrent bleeding, and 44% death within 30 days [12]. 30% of the deaths were related to recurrent bleeding [12]. The use of NBCA-MS embolization was found to be safe and effective with a faster administration time than microcoil [12,17].

In 2017, Torcia et al. explored the use of the Squidperi 18 formula (Balt S.A.S, Montmorency, France) during embolization to manage RSH [19]. Squidperi 18 is a nonadhesive liquid embolic agent [19]. Prior to Torcia et al., Squidperi has been mostly used for arteriovenous malformations, arteriovenous fistulas, aneurysms, and tumors, and not much literature, if any, is available on its uses for RSH [19]. The case presented a patient with a 12x9 cm RSH and two contrast extravasations on a CT angiogram [19]. Squidperi 18 was found to be safe and effective in embolizing the patient’s right epigastric artery despite requiring 20 minutes of formula prep time [19].

Surgical intervention

Surgical intervention has been suggested as a second-line treatment after embolization due to its bleeding risks [9,18]. Surgery arguably may decrease internal hematoma pressure, thereby losing its compression on the bleeding and causing exacerbation of blood loss [9,18]. When embolization cannot control the bleeding, surgery should be considered [18]. Alternatively, surgery can be a first-line treatment for unstable intra-abdominal pressure in situations like compartment syndrome [9]. In McBeth et al., the author examined an abdominal compartment syndrome case due to RSH exacerbations. RSH was initially recognized with a CT scan followed by an anticoagulation reversal and fresh frozen plasma, but the patient’s condition declined with an intraabdominal pressure of 40-60 mm Hg [15]. The hematoma was evacuated surgically with no complications [15]. Intra-abdominal pressure may be a reliable predictor for surgical intervention [15].

Other studies evaluated emergency ligation techniques of the inferior epigastric artery [14,21]. Using ligation techniques, Mantelas et al. found immediate hemodynamic stabilization and suggested surgical ligation as a great alternative to the time-consuming embolization [14]. Another case by Yun et al. argued surgical ligation followed by evacuation of RSH as a quick and simple alternative to embolization [21].

One case found combinational approaches of embolization followed by surgical evacuation to be preferable over surgical ligation [10]. Embolization prior to surgery could halt the bleeding without removing compression, allowing the tamponade effect on the hematoma to take place and decrease the risk of blood loss [10]. However, the combinational approach will require the coordination of hospital teams across multiple units, which may not be feasible in smaller hospital settings [10].

Discussion

This comprehensive literature review on RSH management aims to consolidate current literature into a standardized algorithm to guide past and modern management of spontaneous RSH. Certain predictors are selectively incorporated in the algorithm based on the author’s opinion. An organized consolidation for RSH management is outlined in Figure 1. Because a majority of the cases and studies reviewed are retrospective in nature, generalization of the algorithm is limited, and the algorithm should be referenced by clinicians at their discretion depending on each patient case. Further study on anticoagulant predictors is needed.

## Conclusions

The best management of RSH is dependent on early RSH recognition and implementation of conservative management. In the case of failed CM, predictors such as hematoma size, rate of hemoglobin drop, and the number of red blood cell units transfused are used to guide the best intervention of choice, which may be any of the various types of IR embolization or surgical intervention. Each intervention has its pros and cons and will be subjected to the provider’s discretion depending on the urgency of the individual cases. We hope the consolidated algorithm on current literature can help promote the standardization of protocol in the future. Further research into the best intervention method as dependent on predictors may vary by situation and require further investigation to standardize for RSH use.
